# The Faroese IBD Study: Incidence of Inflammatory Bowel Diseases Across 54 Years of Population-based Data

**DOI:** 10.1093/ecco-jcc/jjw050

**Published:** 2016-03-01

**Authors:** Turid Hammer, Kári R. Nielsen, Pia Munkholm, Johan Burisch, Elsebeth Lynge

**Affiliations:** ^a^Department of Public Health, University of Copenhagen, Copenhagen, Denmark; ^b^Medical Centre, National Hospital, 100 Tórshavn,Faroe Islands; ^c^Genetic Biobank, 100 Tórshavn,Faroe Islands; ^d^North Zealand Hospital, Capital Region, University of Copenhagen, Danish Centre for eHealth and Epidemiology, Copenhagen, Denmark

**Keywords:** Epidemiology, Faroe Islands, inflammatory bowel diseases

## Abstract

**Background and Aims:**

Inflammatory bowel diseases [IBDs] include Crohn’s disease [CD], ulcerative colitis [UC], and IBD unclassified [IBDU]. In 2010 and 2011, the ECCO-EpiCom study found the worldwide highest incidence of inflammatory bowel disease [IBD] in the Faroe Islands: 83 per 100 000 [European Standard Population, ESP]. The present study assessed the long-term time trends in IBD incidence in the Faroese population.

**Methods:**

In this population-based study, data were retrieved from the National Hospital of the Faroe Islands and included all incident cases of CD, UC, and IBDU diagnosed between July 1960 and July 2014. Patients of all ages were included and diagnoses were defined according to the Copenhagen Diagnostic Criteria.

**Results:**

A total of 664 incident IBD patients were diagnosed: 113 with CD, 417 with UC, and 134 with IBDU. Of these, 51 [8%] were diagnosed with paediatric-onset IBD. Between 1960 and 1979, a total of 55 persons were diagnosed; 105 in 1980–89; 166 in 1990–99; 180 in 2000–09; and 158 in 2010–14. This represented an increase in the age-standardised IBD incidence rate from 7, 25, 40, and 42 to 74 per 100 000 [ESP]. For CD, the increase was from 1 to 10, for UC from 4 to 44, and for IBDU from 2 to 21 per 100 000 [ESP].

**Conclusions:**

The high IBD incidence was found to be a relatively new phenomenon. The observed increase is unlikely to be an artefact resulting from, for instance, better registration. Our study indicated a real and increasing disease burden resulting from changing—so far unidentified—exposures.

## 1. Introduction

Worldwide, the incidence of IBD is increasing.^1^ In Europe, 3 million individuals are suffering from IBD and globally the number has reached upwards of 5 million individuals.^2^ IBD is a chronic disease with great individual costs due to an often early onset, changeable disease course, and no known cure. The societal and health care-related expenditures are also substantial.^3^ IBD is believed to derive from a combination of genetic susceptibility and environmental exposures, but the reason for the changing incidence is unknown. Industrialisation, an increased awareness of IBD, and improved diagnostic methods and access to medical services are all possible explanatory factors.^1^

In Europe, differences in the incidence of IBD have been found between the north and south, with a tendency of increasing rates in the south and a plateauing of the higher northern rates.^4^ Furthermore, a west-east gradient of 2 in incidence has been identified.^5^ The European Crohn’s & Colitis Organisation’s Epidemiological Committee study [ECCO-EpiCom]^5,6,7^ found the highest incidence of IBD in the world in the Faroe Islands. The incidence in the Faroe Islands was 83 per 100 000 person-years [py] in the European Standard Population [ESP], whereas it ranged between 4 [Chisinau, Moldova] and 40 [Linköping, Sweden] in the rest of Europe.^5^

The Faroe Islands constitute a unique and well-defined population for the study of IBD and its genetic and environmental risk factors. Complete genealogical, as well as detailed clinical, data from national patient registries are available for the entire population. The Faroese IBD study has been initiated in order to describe the occurrence and clinical presentation of IBD, as well as to search for genetic and environmental risk factors in a homogenous, high-incidence population. In this study, using this unique data source, we investigated the long-term time trend leading to the presently high IBD incidence, as well as the evolution of disease classification in the Faroe Islands.

## 2. Material and Methods

### 2.1. Faroe Islands

The Faroe Islands are an archipelago located in the North Atlantic Ocean between Norway and Iceland, with a total population of 49 000 and with home rule within the Danish Realm. The capital, Tórshavn, has a population of 20 000. The Faroe Islands has a high-quality health care system financed through statutory health insurance and taxes, though with patient charges for selected pharmaceuticals and treatments. The National Hospital in Tórshavn was founded in 1924 and enlarged in the 1960s. Both gastroenterology and abdominal surgery, including colonoscopy, have been specialties at the National Hospital since 1973, with the exception of the period March 1998 to August 2001, when the hospital had no gastroenterologist. In the early survey period, the diagnoses were made either by X-ray or postoperatively and confirmed by a histopathologist in Denmark. At present, the hospital employs one gastroenterologist [KRN], one gastroenterology nurse, and two abdominal surgeons. Since 2011 all general practitioners and hospitals have used the electronic patient record system, *Cosmic*. Patients requiring magnetic resonance [MR] or capsule endoscopy are referred to Hvidovre Hospital and Gentofte Hospital in Denmark. These examinations are usually performed within 1–2 months. Both hospitals send the results as letters to the Faroe Islands, which are scanned into *Cosmic*.

### 2.2. IBD population in the Faroe Islands

An incident case was defined as the date when the diagnostic criteria were met. Data on incident IBD cases are available since 1960^8,9^ and all patient files have been entered into *Cosmic*. Incident cases from 2010 onwards have furthermore been entered into the European Crohn’s and Colitis Organisation’s Epidemiology Committee Database [ECCO–EpiCom Database].^10^ Data were collected retrospectively until 2009 and prospectively from 2010 onwards. The original patient files and all diagnostic criteria were scrutinised in each case by author KRN as a guarantee for the reliability of the data collection. The National Hospital of the Faroe Islands did not have a gastroenterologist for a period of 3 years from 1998 to 2001, but it functioned as a general hospital, including gastro-surgeons. Patients in need of further diagnostics were sent to Denmark. All diagnostic information, including information from Denmark, was collected in the patient files. These files formed the basis for the data being used in the present study.

For the purpose of the present study, data on all incident IBD cases were retrieved from the Diagnosis Registry at the Faroese Genetic Biobank within the Public Health Sector of the Faroe Islands.^11^ The following codes from the 8th and 10th versions of the International Classification of Diseases [ICD] were used for CD: 563.00–563.09 and K50-K50.9. For UC, the following codes were used: 563.19, 569.04, and K51-K51.9. All incident IBD cases diagnosed between July 1960 and July 2014 were retrieved and all were defined according to the Copenhagen Diagnostic Criteria.^12,13^ Unique serial numbers, date of diagnosis, gender, age at onset, date of birth, diagnosis type, date of symptoms, and smoking status were retrieved and clinical characteristics at the time of diagnosis were recorded according to the Montreal classification.^14^ Furthermore, an audit of incident IBD cases was performed [JB] in 2010 and 2011 within the ECCO-EpiCom study. Paediatric-onset IBD was defined as occurring in patients aged 18 years or younger.^15^

### 2.3. Analysis

Incident IBD cases were divided into those of Crohn’s disease [CD], ulcerative colitis [UC], and IBD unclassified [IBDU]. Incident cases were furthermore tabulated by gender and 10-year calendar periods [1960–69, 1970–79, … 2010–14]. The average age at onset, cases per 5-year age group, smoking status, and median time to final diagnosis were calculated. Furthermore, the numbers of CD, UC, and IBDU patients with paediatric-onset IBD were calculated, including the Montreal classification for CD and UC patients.

For the entire period, the age at diagnosis [A], disease location [L], and behaviour [B] for CD patients were calculated as percentages, including disease extent [E] for UC patients. L, B, and E were divided into the following groups: 1960–79, 1980–89, 1990–99, 2000–09, 2010–14. To test the null hypothesis that the distribution of L was the same over calendar time against the alternative hypothesis, that there were differences, a Fisher’s exact test was performed. The same test was carried out for B, whereas the corresponding set of hypotheses for E were tested using a Pearson’s chi-square test due to a larger sample size. A level of 5% was set for statistical significance.

Age-standardised incidence rates were calculated for CD, UC, IBDU, and all IBD by calendar period using the ESP. Due to the small numbers for 1960–69 and 1970–79, numbers for these years were merged, and the age-standardised rates calculated for 1960–79, 1980–89, 1990–99, … 2010–14. Incidence rates were also stratified by age and gender for the period 1960–2014.

We tabulated incidence rates specified by 5-year age groups [0–4, … 85–89] and 5-year calendar periods [1960–64, … 2010–14]. Based on these rates we calculated the cumulative risk for some selected birth cohorts from ages 15 to 50 years, and for some other birth cohorts from ages 40 to 75 years.

Person-years [py] were estimated based on Faroese population data for 1960–84 retrieved from Statistics Denmark [dst.dk] and for 1985-2014 from Statistics Faroe Islands [hagstova.fo]. Statistical analyses were performed using Stata software, version 13.1 [Stata, College Station, TX, USA].

### 2.4. Ethics

This is a register-based research project with no contact with patients, their relatives, or treating physicians. In order to obtain the anonymised data, we [TH] received clearance from the Medical Centre at the National Hospital of the Faroe Islands and from the Faroese Genetic Biobank. All authors had access to the study data and have reviewed and approved the final manuscript.

## 3. Results

A total of 664 persons were diagnosed with IBD between 1960 and 2014 [Table 1]. The number of incident cases increased by a factor of 10, from 17 in the first decade [1960–69] to 180 in the last decade [2000–09], and to 158 in the half-decade 2000–14.The incident cases were almost equally divided between men and women. UC dominated with 417 out of the 664 cases, whereas CD included 113 cases, and 134 cases were registered as IBDU. One patient was excluded due to incomplete electronic registration and loss of original file. Paediatric-onset IBD included 29 UC patients of whom three were classified with E1, seven with E2, and 19 with E3. Of the 13 paediatric CD patients, disease location was L1 for three, L2 for six, L3 for three, and L3 + L4 for one patient. Behaviour was B1 for eight patients, B2 for three, B3 for one, and B3 perianal (B3p) for one. With IBDU, nine patients were 18 years old or younger.

**Table 1.** Demographic and clinical characteristics of IBD patients from 1960 to 2014.

	Periods						
	1960–9	1970–9	1980–9	1990–9	2000–9	2010–4	1960–2014
Men							
CD	2	1	11	14	23	9	60
UC	5	14	40	52	62	54	227
IBDU	3	5	5	13	12	20	58
Total	10	20	56	79	97	83	345
Women							
CD	1	2	5	12	21	12	53
UC	5	10	37	56	43	39	190
IBDU	1	6	7	19	19	24	76
Total	7	18	49	87	83	75	319
Men & women							
CD	3	3	16	26	44	21	113
UC	10	24	77	108	105	93	417
IBDU	4	11	12	32	31	44	134
Total	17	38	105	166	180	158	664
**Number [%]**			**CD**		**UC**	**IBDU**	**Total**
Total patients			113 [17%]		417 [63%]	134 [20%]	664 [100%]
-Men			60 [53%]		227 [54%]	58 [43%]	345 [52%]
-Women			53 [47%]		190 [46%]	76 [57%]	319 [48%]
Average age at onset, years^a^			41 [10–85]		41 [0–86]	40 [3–82]	41 [0–86]
Cases per age-group							
0–4			0 [0%]		3 [60%]	2 [40%]	5 [100%]
5–9			0 [0%]		2 [50%]	2 [50%]	4 [100%]
10–14			3 [30%]		6 [60%]	1 [10%]	10 [100%]
15–19			14 [29%]		28 [57%]	7 [14%]	49 [100%]
20–24			15 [21%]		37 [52%]	19 [27%]	71 [100%]
25–29			4 [7%]		43 [70%]	14 [23%]	61 [100%]
30–34			13 [15%]		59 [67%]	16 [18%]	88 [100%]
35–39			8 [12%]		45 [67%]	14 [21%]	67 [100%]
40–44			12 [22%]		37 [67%]	6 [11%]	55 [100%]
45–49			7 [13%]		34 [64%]	12 [23%]	53 [100%]
50–54			12 [24%]		29 [57%]	10 [20%]	51 [100%]
55–59			5 [14%]		18 [50%]	13 [36%]	36 [100%]
60–64			4 [14%]		22 [73%]	4 [14%]	30 [100%]
65–69			6 [24%]		17 [68%]	2 [8%]	25 [100%]
70–74			3 [10%]		18 [62%]	8 [28%]	29 [100%]
75–79			4 [21%]		13 [68%]	2 [11%]	19 [100%]
80–84			2 [22%]		5 [56%]	2 [22%]	9 [100%]
85–89			1 [50%]		1 [50%]	0 [0%]	2 [100%]
* N*			[113]		[417]	[134]	[664]
Smoking status at time of diagnosis							
-Non-smoker			43 [46%]		170 [51%]	40 [45%]	253 [49%]
-Current smoker			35 [36%]		68 [20%]	25 [28%]	128 [25%]
-Former smoker			17 [18%]		97 [29%]	24 [27%]	138 [27%]
*N* ^b^			[95]		[335]	[89]	[519]

^a^Data on date of symptoms onset were available for 658 patients.

^b^Data on smoking were available for 519 patients, as these data were normally not available for patients diagnosed before 1990.

**Figure 1.**
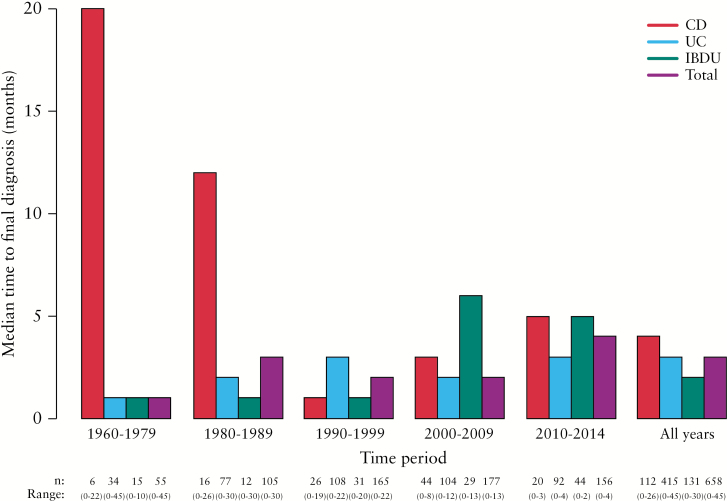
Median time to final diagnosis.

The age at onset varied from early childhood to old age [range from 0 years to 86 years], the average age at onset was 41 years for CD and UC, and slightly younger for IBDU [Table 1]. The median time to final diagnosis was 4 months for CD, 3 months for UC, and 2 months for IBDU. The median time to final diagnosis was divided into calendar periods [1960–79, 1980–89, … 2010–14]. For CD this ranged from 20 months in 1960–79 to 5 months in 2010–14. A slight upward trend during the most recent decades was not found to be significant [*p* = 0.39]. The time for UC ranged from 1 month in 1960–79 to 3 months in 2010–14. For IBDU, the median time to final diagnosis was 1 month in 1960–79 and increased to 5 months in 2010–14 [Figure 1]. Recent changes were not found to be significant [*p* = 0.07].

Regarding smoking status at time of diagnosis, 46% of the CD patients were non-smokers at time of diagnosis, 36% current, and 18% were former smokers. For UC patients the proportions were 51%, 20%, and 29%, respectively. For IBDU patients, the proportions were 45%, 28%, and 27%, respectively [Table 1].

**Figure 2.**
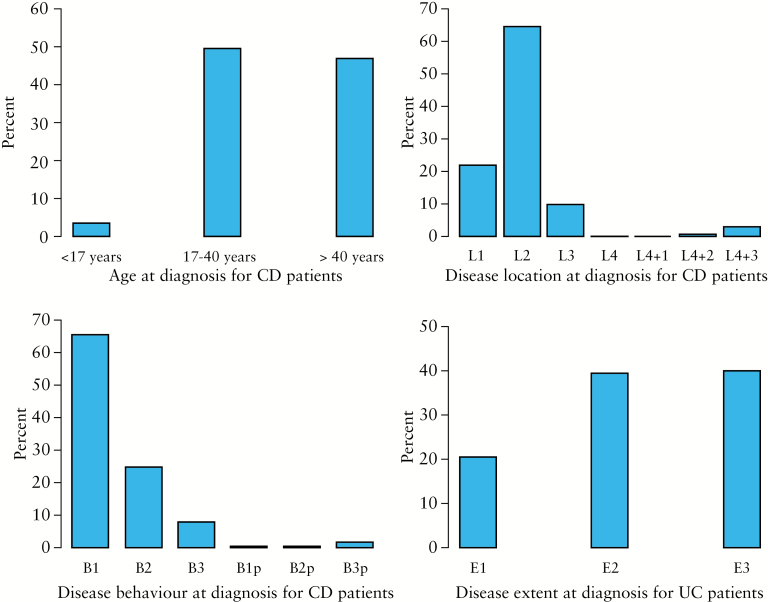
Montreal classification for Crohn’s disease [CD] and ulcerative colitis [UC] patients from 1960 to 2014.

For the period of 1960–2014, age at diagnosis [A] for CD patients was distributed as 50% [*N* = 56] aged 17–40 years, 46% [*N* = 53] were over 40 years, and 4% [*N* = 4] were below 17 years. Disease location [L] for 65% [*N* = 73] of the CD patients was classified as L2, 22% [*N* = 25] as L1, 10% [*N* = 11] as L3, 3% [*N* = 3] as L3 + L4, and 1% [*N* = 1] as L2 + L4. Behaviour [B] at diagnosis for CD patients was for 65% [*N* = 74] classified as B1, 25% [*N* = 28] as B2, 8% [*N* = 9] as B3, and 2% [*N* = 2] as B3p. Regarding extent [E] in UC, 40% [*N* = 167] were classified as E3, 39% [*N* = 164] as E2, and 21% [*N* = 86] as E1 [Figure 2]. Period-specific numbers were small with considerable random variation. Fisher’s exact test for L and B and Pearson’s chi-square test for E were performed and all three tests were highly significant [*p*-value < 0.001], ie the distribution of L, B, and E was not the same across calendar periods [data not shown].

**Figure 3.**
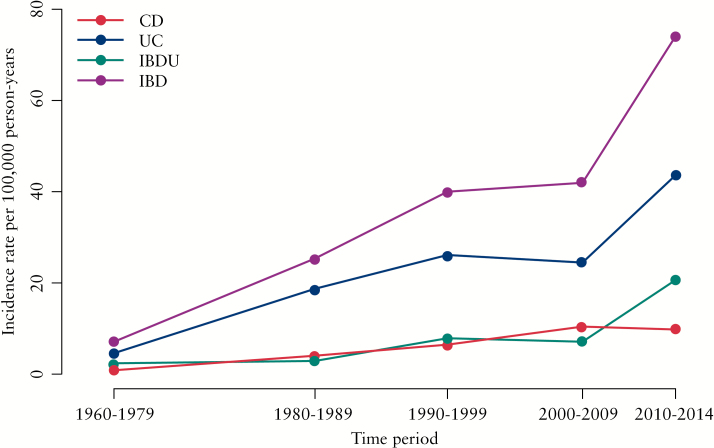
Inflammatory bowel disease [IBD] age-standardised European Standard Population [ESP] incidence rates per 100 000 person-years [py].

CD was almost non-existent in 1960–79 [Figure 3], but increased to about 10 per 100 000 [ESP] from the year 2000 onwards. UC was at a level of 4 per 100 000 in 1960–79 and increased up to 26 per 100 000 in 1990–99, where it remained for the next 10 years before increasing dramatically to 44 per 100 000 between 2010 and 2014. A rapid increase during this most recent 5-year period was also observed for IBDU. In total, IBD incidence was about 7 per 100 000 in the Faroe Islands in 1960–79, rising to 74 per 100 000 in 2010–14.

**Figure 4.**
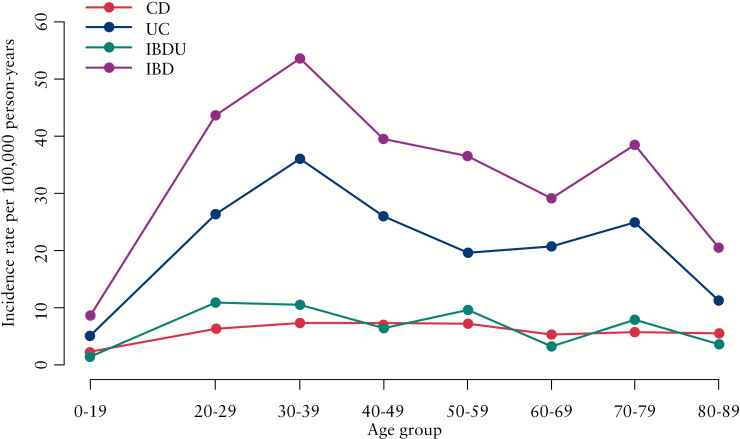
Incidence rates per 100 000 person-years [py] for age groups from 1960 to 2014.

**Figure 5.**
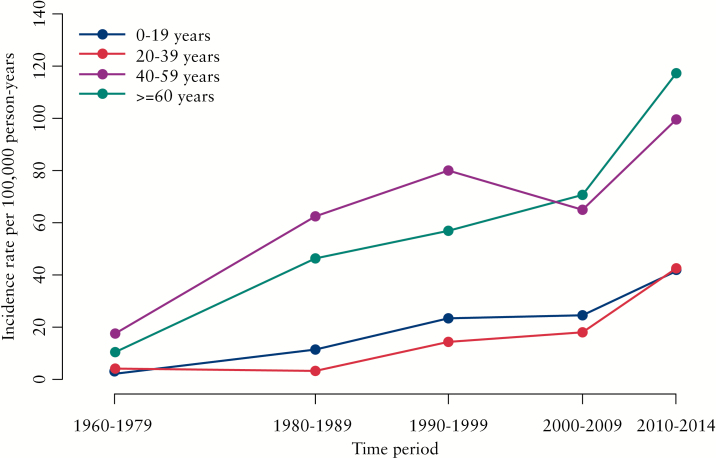
Incidence rates per 100 000 person-years [py] for age segments over time periods.

**Figure 6.**
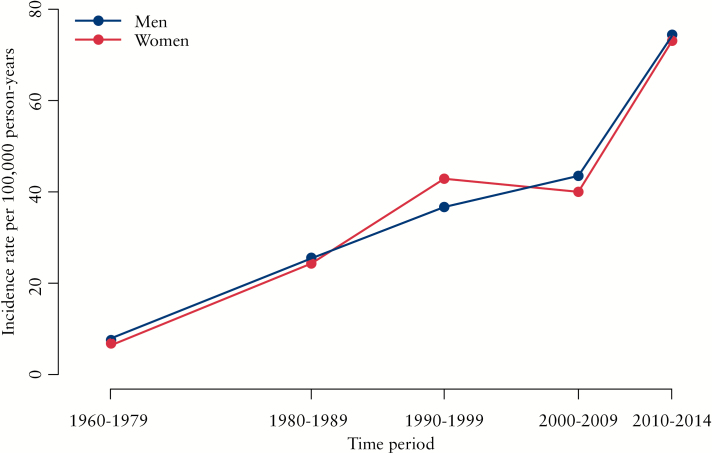
Incidence rates per 100 000 person-years [py] for men and women from 1960 to 2014.

The incidence was divided into age groups, revealing a peak around age 30–39 years and a second, smaller, peak around age 70–79 years [Figure 4]. Distributing the incidence according to age-segments and five time periods shows a difference in the younger vs the older age groups, with IBD highest among the older participants throughout the study period [Figure 5]. Furthermore, the incidence was fairly similarly distributed when stratified by gender [Figure 6].

The cumulative risk for IBD from age 15 years to age 50 years was 0.7% for persons born in 1940–49, and increased to 1.4% for persons born in 1960–69. The cumulative risk from age 40 to age 75 years was 0.7% for persons born in 1915–24, and increased to 1.0% for persons born in 1930–39.

## 4. Discussion

The Faroese population has experienced a dramatic increase in the incidence of IBD, especially of UC. A more than 7-fold increase was observed in IBD incidence from the beginning to the end of the 54-year period covered by the present study. Two important observations are notable: first, the record high incidence of IBD observed in the ECCO-EpiCom study in 2010 and 2011 was confirmed when data from the years preceding 2010–14 were included in the calculation; and second, the Faroese population has not always been at high risk of IBD. The current high incidence is a phenomenon that has developed gradually over half a century, and it affected persons of both young [15–50 years] and older [40–75 years] ages. These observations stress the uniqueness of the Faroese IBD study.

### 4.1. True increase or artefact?

Given IBD data such as those observed in the Faroe Islands, the question inevitably rises as to whether these data reflect a true change in the underlying burden of disease or whether they represent an artefact due to better diagnostics and/or registration. In the following we will address these questions by examining possible changes in health care facilities, registration of patient records, and health care behaviour.

Being part of the Danish Realm, health care in the Faroe Islands has always been comparable to the level of health care in northern Europe. By far the majority of its physicians have graduated from Denmark. Patient files in paper form have been stored at the hospital since 1960. The decreasing trend in the median time to final diagnosis for CD is similar to observations reported elsewhere.^16^ This could suggest that the increased incidence might partly be due to earlier diagnosis with a shorter interval between onset of symptoms and diagnosis. For UC patients, the median time to final diagnosis was low and stable throughout the period. It is unlikely that changes in health care facilities and/or registration of patient records alone could explain the increase in the incidence of IBD.

Although an archipelago surrounded by rough seas, communication between the islands has always been substantial. The first ferry started operation in 1949. The first tunnel was built in 1963, and the islands are now connected via 19 tunnels. Changes in the infrastructure have thus made access to health care easier, but it is unlikely that this alone could explain the observed increase in the IBD incidence. Furthermore, although increased diagnostic awareness could have played a role, as indicated by the increased proportion of E1 UC patients, one should note that the educational level has been high—comparable to that of other Nordic countries—throughout the study period.

Novel diagnostic methods and tools were implemented at the same time as in other Nordic countries. Colonoscopy, for instance, was available as early as 1973. Thus we consider the presently record-high IBD incidence to reflect a truly greater risk faced by the Faroese population. Furthermore, we expect this incidence to be a phenomenon that has emerged within the past half century.

### 4.2. Other studies

Worldwide, the incidence of CD is between 0 and 20 per 100 000 and for UC between 0 and 24 per 100 000.^1^ Our data are in line with those from other Nordic countries where UC is more frequent than CD.^1,17^ IBD was previously considered a disease of Western countries, but more recently it has been observed in many places around the world, such as a rapid increase seen in Japan.^18,19^ Worldwide, the incidence of UC appears to have increased first, followed by an increase in CD.^20^ The same pattern was found for the Faroe Islands. A striking finding in our study was the high proportion of IBDU, at 20%, compared with the 5–15% reported from other places.^21^ In the Faroe Islands, an IBDU diagnosis was given when a patient did not fulfill the Copenhagen Diagnostic Criteria completely, but was in need of IBD therapy. The diagnosis is often considered an initial and temporary diagnosis leading to a later diagnosis of CD or UC.^21^ All patient files were thoroughly reviewed by author KRN to ensure that an IBDU diagnosis was not a case of either CD or UC within the first 3 months after the diagnosis.

The Faroese patients also had a higher average age at onset of 41 years for CD and UC and 40 years for IBDU, than found elsewhere.^6^ The stratification of incidence according to age showed that IBD is prominently occurring in the second to fourth decades of life. This trend is consistent with findings in other studies.^1^ The proportion of L1 and L3 CD patients was 32%, which was lower than that seen in other settings.^3,5^ The higher proportion of L2 patients at 65% therefore differentiates this from other studies.^5,18^ We have no explanation and no hypothesis for this finding. The low proportion of perianal disease among CD patients found in this study differs from other findings, although perianal disease has scarcely been mentioned in population-based studies.^22,23^ A plausible explanation could be the available diagnostic methods in the early period of the study period. However, the findings in this study are in line with the findings from the 2010 and 2011 EpiCom cohorts, in which the diagnostic methods had evolved substantially.^5,6^

Although the clustering within families has been well documented,^24,25^ in Denmark first-degree family members only had a 6-fold higher incidence than among the general population,^26^ suggesting an important environmental component in the aetiology. This finding is further substantiated by the fact that migration from low-risk to high-risk areas is associated with an increase in incidence.^18,27^ A Swedish study found incidence to depend both on country of origin and on status as first- or second-generation immigrant.^28^ The incidence of IBD is also high on Iceland: 29 per 100 000 [ESP] in 2010.^5^ The population of Iceland resembles the population of the Faroe Islands in terms of frequent family relatedness. The genetic composition and shared environmental risk factors are believed to play a major role in the development of IBD in Iceland.^29^

The hygiene hypothesis states that a Western lifestyle with reduced family size, vaccinations, high antibiotic usage, improved sanitation, clean drinking water, and the adoption of Western dietary habits all contribute to the growing burden of allergic and autoimmune diseases in general.^30^ IBD incidence has been found to correlate with upbringing in rural vs urban environment, helicobacter pylori, family size and birth order, the enteric microbiota, appendectomy, oral contraceptive pills, and stress.^31,32^ Smoking has been found to be protective for UC and a risk factor for CD.^33^ Vitamin D deficiency in IBD patients is well known, but the role of vitamin D insufficiency as contributing to, or as a manifestation of, IBD remains unclear.^34^ Differences in diet are considered to be the most topical and likely factors in explaining the geographical variation and rising incidence,^17^ although the role of specific foods remains inconclusive.^35,36^

### 4.3. What could explain the rising IBD incidence in the Faroe Islands?

The Faroese population descends from Vikings, with the first settlement dating back to around 825–75 and a high level of asymmetry, with more men of Scandinavian ancestry and more women of Scottish and Irish ancestries. The population expanded slowly from 4000 inhabitants in the late 1300s, to 5000 in 1800, followed by an increase to the current 49 000 inhabitants. As the Faroese population has been isolated and homogenous, genetic drift is assumed to have been of great importance for the present gene pool.^37,38^ Genetically inherited diseases are common, eg carnitine transporter deficiency and holocarboxylase synthase deficiencies,^39^ glycogen storage disease type IIIA,^40^ cystic fibrosis,^41^ bipolar affective disorder,^42^ and schizophrenia.^43^ IBD has also been found to aggregate in families in the Faroe Islands. An old Faroese study found 9.4% of the participants to have relatives with an IBD.^8^ However, the genetic composition of the Faroese population is expected to have remained fairly constant over the past 50 years. Familial predisposition alone can therefore not explain the dramatic increase in IBD incidence over time, which is more likely to be explained by changes over time in environmental risk factors.

Westernisation during the past 50 years has clearly changed the life of the Faroese population. The employment structure has changed, with fewer people making a living from farming and/or fishing, housing has evolved with better heating and facilities, and diet has changed from the traditional fish, lamb, and pilot whale meat to include a broader variety of imported foods. Today the Faroese Food and Veterinary Authority recommends that adults consume one meal of pilot whale and blubber per month at most, females who are planning to become pregnant should not consume blubber, and women who are pregnant or breastfeeding should refrain from consuming pilot whale meat. The registration of smoking status was not complete before the 1990s. Although smoking has decreased, the Faroe Islands still had the highest percentage of daily smokers in 2012 among the Nordic countries, at 27% for men and 28% for women.^44^ In-depth studies are needed to pinpoint specific risk factors associated with the high IBD incidence. Later phases of ‘The Faroese IBD Study’ will focus on investigating characteristics of IBD patients, addressing familial disposition, dietary factors, the microbiota, and environmental exposures.

### 4.4. Conclusion

The Faroe Islands presently have the highest reported IBD incidence in the world. In the present study, covering the past 54 years, we found this high incidence to be a relatively new phenomenon emerging in the 1960s onwards. Although improved health care, better transportation and access to health care, and increased disease awareness might all have played a role, these factors alone are unlikely to explain the observed increase in IBD incidence. The increase in IBD incidence is therefore assumed to be a real increase in the disease burden resulting from changing, though as yet unidentified, exposures.

## Funding

This work was supported by the European Crohn’s and Colitis Organisation [ECCO], and the Beckett Foundation [to TH].

## Conflict of Interest

None.

## Author Contributions

All authors contributed to this work. KRN collected the data, TH drafted the manuscript, JB, PM, EL, KRN, and TH analysed the data and revised the manuscript.

## Abbreviations

CD: Crohn’s disease;
IBD: inflammatory bowel disease;
IBDU: inflammatory bowel disease unclassified;
UC: ulcerative colitis.
